# Continuous light at fixed daily light integral enhances lettuce growth due to improved light interception and light use efficiency

**DOI:** 10.3389/fpls.2026.1756524

**Published:** 2026-02-03

**Authors:** Diego Núñez Ocaña, Homa Esmaeli Sooderjani, Paul Kusuma, Leo F. M. Marcelis, Ep Heuvelink

**Affiliations:** 1 Horticulture and Product Physiology, Wageningen University & Research, Wageningen, Netherlands; 2 Agricultural Biosystems Engineering, Wageningen University & Research, Wageningen, Netherlands

**Keywords:** continuous light (CL), controlled environment agriculture, energy-use efficiency (EUE), lettuce (*Lactuca sativa*), light use efficiency (LUE), photoperiod, vertical farming

## Abstract

Vertical farming (VF) allows precise control of growth conditions, but its high energy demand underscores the need to improve light-use efficiency. This study examined how continuous lighting (CL) affected the growth, morphology, and carbohydrate content of lettuce cultivars ‘Jagger’ and ‘Danstar,’ compared with the 18-hour photoperiod at the same daily light integral. The study also modelled the influence of photon efficacy of the LEDs under both lighting regimes on energy use and energy-use efficiency (EUE). Under CL, leaf area growth increased total light interception by 10% in ‘Jagger’, whereas in ‘Danstar’ it remained similar to the 18-hour photoperiod. Carbohydrate metabolism shifted, with sucrose decreasing and starch accumulating under CL, while other sugars remained unchanged. CL improved the use efficiency of intercepted light (LUE) by 7 to 11% relative to the 18-hour photoperiod. Consequently, fresh and dry mass increased by 23% in ‘Jagger’ and by 6% and 14% in ‘Danstar,’ respectively. Assuming the same photon efficacy of the LEDs, CL required the same energy input as the 18-hour photoperiod; however, due to the growth enhancement under CL, it resulted in a 16 to 18% higher EUE. Visual quality was unaffected in both cultivars, with no signs of tip-burn or bolting. These findings indicate that CL can simultaneously improve lettuce growth and energy use efficiency, offering a promising strategy to reduce energy costs and lighting investment in vertical farming.

## Introduction

Vertical farming (VF) enables precise control of the growth environment, including lighting and climate, which are essential for optimal plant growth (Mitchell, 2022; van Delden et al., 2021). Greenhouses also optimise such control, but the utilisation of such technology is higher in vertical farms (Graamans et al., 2018; Kozai and Niu, 2016). The economic viability of VF largely depends on optimising lighting and climate control to maximise yield, improve energy and light use efficiency (LUE, which is the biomass—fresh or dry—produced per unit of incident light), and ensure high product quality (Asseng et al., 2020; Eichelsbacher et al., 2025; Jin et al., 2023a).

Lettuce (*Lactuca sativa*) is one of the most widely consumed leafy vegetables worldwide, with high demand in fresh markets and ready-to-eat products, and is among the most extensively researched and cultivated crops in VF (Boros et al., 2023; Fallovo et al., 2009; Viacava et al., 2018). Modern lettuce varieties are classified according to their morphological characteristics (Wei et al., 2021), with ‘leaf lettuce’ type being particularly suitable for VF and high-tech greenhouse cultivation. We selected two phenotypes of this leaf lettuce type for this study: ‘Danstar’, characterised by slower growth and curly, crisp, upright leaves, and ‘Jagger’, which exhibits faster growth and softer, flat, horizontally oriented leaves.

In VF, the light environment can be customised by: 1) photosynthetic photon flux density (PPFD), which represents the radiation in the 400 to 700 nm range, although other wavelengths such as far-red radiation (700 to 800 nm) can also influence growth and morphology; 2) photoperiod, the duration of radiation exposure; and 3) spectral composition, referring to the relative proportions of different wavebands, such as blue, green, red, and far-red, which is beyond the scope of the present study. PPFD and photoperiod together determine the daily light integral (DLI), which is highly correlated with biomass accumulation (Poorter et al., 2019). Extending the photoperiod to continuous light (CL) allows PPFD to be reduced to the minimum possible level to reach a desired DLI. This may potentially lower energy consumption for several reasons. First, low PPFDs can be the result of a decreased drive current density within the LED fixture, and this increases the photon efficiency (Kusuma et al., 2020). Second, plants at low PPFD may experience a higher quantum yield (efficiency of carbon capture per photon) (Elkins and van Iersel, 2020b; Mayorga-Gomez et al., 2024). Third, extension of the photoperiod up to CL could enhance canopy size and optimise light interception. At the same DLI, extending the photoperiod from 10- to 21-hours led to a linear increase in canopy size and light interception in lettuce and rudbeckia (Elkins and van Iersel, 2020a; Palmer and van Iersel, 2020). During the photoperiod, cooling is necessary to remove the heat generated by lamps and other electrical equipment (Kozai, 2018). At lower PPFD, less radiant energy is supplied to the VF system per unit time, which in turn reduces heat generation and thus lowers the cooling demand of HVAC systems. Overall, these responses indicate that CL with a low PPFD would be the most efficient condition for production.

The plant circadian clock regulates biological rhythms that optimise growth, photosynthesis, leaf movement, and non-structural carbohydrate dynamics (Dodd et al., 2005; Kim et al., 2017; Steed et al., 2021). Although CL is feasible in VF, its application is constrained by disruption of the dark period and the loss of diel cues such as sunrise and sunset, which are essential for proper circadian function (Marie et al., 2022). In several species, CL has been associated with chlorosis, reduced growth, leaf deformation, carbohydrate imbalance, and photoinhibition (Matsuda et al., 2014; Shibaeva et al., 2023; Velez-Ramirez et al., 2011). These effects are commonly linked to circadian misalignment, which can impair starch turnover and carbohydrate allocation, either through excessive sugar accumulation in source leaves or through incomplete starch degradation before dawn, ultimately limiting carbon availability for growth (Graf et al., 2010; Tanigaki et al., 2019; Velez-Ramirez et al., 2011).

Lettuce appears generally tolerant of CL, though some cultivars exhibited increased tip-burn compared to a 16-hour photoperiod (Koontz and Prince, 1986). At relatively low DLIs (10 or 13 mol·m¯²·d¯¹), CL had little effect on lettuce fresh and dry mass compared to a 16-hour photoperiod. At a higher DLI of 15.6 mol·m¯²·d¯¹, CL increased fresh mass by ~20% and dry mass by ~23% compared to the 16-hour photoperiod (Kelly et al., 2020). Núñez Ocaña et al. (2025) observed no carbohydrate rhythms under CL, and no signs of chlorosis or necrosis. Moreover, visual inspection indicated no adverse effects of CL on the growth of lettuce cultivars ‘Jagger’ and ‘Danstar’ compared with the 18-hour photoperiod. Conversely, at a fixed PPFD, CL (22 mol·m¯²·d¯¹ DLI) decreased lettuce fresh mass by 18% while increasing dry mass by 26% compared to the 16-hour photoperiod (14 mol·m¯²·d¯¹ DLI) (Pennisi et al., 2020a). It is unclear whether the observed fresh mass reduction (without chlorosis or necrosis) was caused by excessive DLI or by circadian clock disruption.

Under equal DLI, exposure to relatively lower PPFD during extended photoperiods (near CL) has been associated with reduced specific leaf area, indicating thicker or denser leaves (Elkins and van Iersel, 2020a; Yan et al., 2021) and higher chlorophyll content compared to higher PPFD during shorter photoperiod (Palmer and van Iersel, 2020). Although leaves grown under low PPFD exhibit a lower photosynthetic rate than those under higher PPFD (Warner et al., 2023), their PSII efficiency is relatively higher (Elkins and van Iersel, 2020b). On the contrary, leaves grown under higher PPFDs contain more rubisco and anthocyanins, and longer palisade cells (thicker leaves) (Taiz and Zeiger, 2010; Vogelmann and Martin, 1993). In *Arabidopsis thaliana* (wild types), leaf area growth typically fluctuates through the day, regardless of photoperiod length (8-, 12-, or 16-hours) or cycle lengths (days with 17-, 24-, or 28-hours; sum of photoperiod and dark period). CL suppressed such diurnal fluctuations and led to a slightly greater average leaf area growth during the photoperiod than during the corresponding subjective dark period. A mismatch between the circadian clock and the cycle length resulted in earlier and stronger inhibition of leaf area growth during the last hours of the dark period (Apelt et al., 2017), which was also associated with incorrect starch catabolism (Graf et al., 2010). Limited floor coverage decreases VF profitability by reducing canopy light interception, resulting in less canopy photosynthesis and, consequently, less plant growth (Klassen et al., 2004).

Energy use efficiency (EUE) refers to the amount of fresh or dry mass produced per unit of energy consumed for lighting, climatisation or both. At a fixed 16-hour photoperiod (unequal DLI), EUE was shown to have an optimal response to PPFD, peaking between 200 and 250 µmol·m^-2^·s^-1^, which coincided with optimal growth in this PPFD range (Pennisi et al., 2020b). Conversely, at 250 µmol·m^-2^·s^-1^ lettuce EUE (based only on LED energy use), linearly decreased when the photoperiod was extended from a 16-hour photoperiod to CL (Pennisi et al., 2020a). In lettuce, Chen et al. (2022) reported that energy use efficiency increased with longer cycle lengths (24 h → 180 h) under an equivalent light sum. These results may suggest that extending the duration of the cycle length may promote the conversion efficiency of incident energy into plant dry mass.

We aimed to investigate the effect of CL on lettuce growth, morphology, carbohydrate content, and energy use. We investigated if CL reduces leaf area growth because this process mainly occurs during the dark period, resulting in reduced light interception. Moreover, we researched whether CL results in lower LUE and growth compared with the 18-hour photoperiod. Additionally, we studied if at equal DLI, CL disturbs carbohydrate metabolism due to sustained assimilation and the absence of a dark period for starch catabolism, resulting in reduced growth.

## Materials and methods

The experiment was conducted in a climate chamber divided into twenty-one lightproof growth compartments (Dimensions: 152 × 54 × 75cm L x W x H), organised in three stacked rows. Per experimental replicate, we randomly selected two growth compartments for germination and two for the corresponding treatments (see section: Light treatments). Inside each growth compartment, air temperature and relative humidity were recorded at 5-minute intervals using a radiation-shielded temperature and relative humidity sensor and data logger (HOBO MX2301A; Onset Computer Corporation, Bourne - USA). Throughout the experiment, the average air temperature was 23 ± 0.4°C, with a relative humidity of 81 ± 2% (VPD = 0.54 kPa), regardless of photoperiod, dark period, or treatment (**Supplementary Figure S1**). The horizontal airflow (at leaf level) within each lightproof compartment ranged from 0.3 to 0.6 m·s^-1^, measured with a 3D-axis anemometer (WindMaster ultrasonic; Gill Instruments, Lymington - UK). Carbon dioxide (CO_2_) was enriched to an average concentration of 815 ± 14 ppm measured with a CO_2_ sensor (Vaisala Carbon Dioxide Probe GMP252; Vaisala Oyj, Helsinki - Finland).

### Plant material, germination and transplant

Unprimed and uncoated seeds from *Lactuca sativa*, cultivars ‘Danstar’ and cv. ‘Jagger’ (Nunhems Netherlands BV, Nunhem - The Netherlands) were sown in germination trays with stone wool plugs (Grodan plantop plug NG2.0; Grodan, Roermond - The Netherlands) and vernalised in darkness for three days at 4°C. Next, the germination trays were transferred to the growth compartments. Seedlings received 200 ± 11 µmol·m^-2^·s^-1^ PPFD during the 18-hour photoperiod via overhead LED modules (GreenPower LED production module deep red/white 150; Philips, Eindhoven - The Netherlands), the spectrum contained 8% blue light (400 to 499 nm), 18% green light (500 to 599 nm), and 74% red light (600 to 699 nm).

Eleven days after sowing, uniform seedlings were transplanted into stone wool blocks (7.5 x 7.5 x 6.5 cm, Grodan delta block; Grodan, Roermond - The Netherlands) and transferred to other growth compartments. Planting density was defined based on centre-to-centre spacing between plants arranged in a chessboard pattern, with 15.6 cm between plant centres and 11.5 cm between rows, corresponding to a density of 56 plants·m^-2^. Stone wool blocks were irrigated via an ebb and flood system that supplied nutrient solution containing: 12.92 mM NO_3_^−^, 0.38 mM NH_4_^+^, 1.53 mM H_2_PO_4_^−^, 8.82 mM K^+^, 4.22 mM Ca^2+^, 1.53 mM SO_4_^2−^, 1.15 mM Mg^2+^, 0.12 mM HCO_3_^−^, 0.38 mM SiO_3_^2−^, 30.67 μM Fe^3+^, 38.33 μM B, 1.53 mM Cl^−^, 3.83 μM Mn^2+^, 3.83 μM Zn^2+^, 0.77 μM Cu^2+^ and 0.38 μM Mo, nutrient formula was according to Jin et al. (2021), and replaced weekly to preserve its formulation and maintain a pH of 5.8 and electrical conductivity of 2.3 dS·m^-1^.

### Light treatments

After transplant (0 DAT), plants were immediately exposed to either 18-hour photoperiod or CL (continuous light). PPFD was supplied via LED modules (GreenPower LED production module deep red/white 150; Philips, Eindhoven - The Netherlands) and far-red radiation (700 to 800 nm) via dimmable LED modules (peak wavelength 730 nm, GreenPower LED Research module Far Red; Philips, Eindhoven - The Netherlands). PPFD and far-red radiation were adjusted for each treatment to maintain the same daily light integral (DLI) of 16.8 mol·m^-2^·d^-1^ or total photon flux density (TPFD) of 20.1 mol·m^-2^·d^-1^ across treatments (**Table 1**). PPFD, far-red radiation, and spectrum were measured at the beginning and end of each experimental replicate, at the centre of the growth chamber and the height of the stone wool block, using a PAR sensor (LI-COR, LI-250A from Lincoln, NE - USA) and a spectroradiometer (Apogee Instruments model SS-110 from Logan, UT - USA). Regardless of PPFD or treatment, the spectrum contained 6.5% blue light (400 to 499 nm), 14.8% green light (500 to 599 nm), 61.3% red light (600 to 699 nm), and 17.4% far-red light (700 to 800 nm) (**Supplementary Figure S2**). Spectrum had a red to far-red ratio (R:FR) of 3.5 and phytochrome photostationary state (PSS) of 0.82, calculated according to Sager et al. (1988).

**Table 1:** Photosynthetic photon flux density (PPFD 400 to 700 nm) and far-red radiation (700 to 800 nm, peak 730 nm) supplied during the 18-hour photoperiod and CL (continuous light).

Treatment	PPFD (µmol·m^-2^·s^-1^)	Far-red (µmol·m^-2^·s^-1^)	PFD* (µmol·m^-2^·s^-1^)	DLI (mol·m^-2^·d^-1^)	TPFD** (mol·m^-2^·d^-1^)	R:FR	PSS
18-hour photoperiod	258±3	50±1	308±3	16.7±0	20.0±0	3.5±0	0.82±0
CL	196±1	38±1	234±1	17.0±0	20.2±0	3.5±0	0.82±0
CL *vs*. 18-hour Phot. % change	-24%	-24%	-24%	1%	1%	0%	0%

Values represent the average ± standard deviation calculated from four independent replicate experiments.

* PFD (photon flux density) = PPFD (400 to 700nm) + FR (700 to 800 nm).

** TPFD (total photon flux density per day) = PFD x photoperiod x 3600 s·h^-1^ ÷ 1×10^6^ µmol·mol^-1^.

PFD (photon flux density, range 400 to 800 nm). Daily light integral (DLI, 400 to 700 nm) and total photon flux density per day (TPFD, 400 to 800 nm). Red to far-red ratio (R:FR) calculated from red (600 to 700 nm) and far-red (700 to 800 nm) wavelengths. Phytochrome photostationary state (PSS) calculated according to Sager et al. (1988).

### Non-destructive measurements

During the first three experimental replicates, RGB cameras (VIZU Extreme X6S Wi-Fi 4K Action Camera - The Netherlands) were installed between the LED modules at 35 ± 1 cm (18-hour photoperiod) and 40 ± 1 cm (CL) above the surface of the light-proof compartment. Automated top-view imaging of lettuce plants was performed daily from 0 to 19 days after transplanting (DAT) with eleven ‘Danstar’ and ten ‘Jagger’ plants per image. Imaging was conducted at the beginning, midpoint (noon) and end of the 18-hour photoperiod or at equivalent times under CL. Image classification and segmentation followed the methodologies of Núñez Ocaña et al. (2025) and Easlon and Bloom (2014).

### Projected leaf area measurements

From 2 to 13 DAT, no leaf overlap was observed between plants, allowing for individual projected leaf area (PLA) measurements. From 2 to 13 DAT, the increase of PLA over DAT was fitted by an exponential function (Equation 1) according to Hobbie and Roth (2007).

(1) $$PLA=PLA_{Initial}\cdot \;e^{\left(RGR_{PLA1}\cdot DAT\right)}$$

The nonlinear least squares (NLS) approach was implemented using the ‘nlme’ package (Pinheiro et al., 2025) in R software (R Core Team, 2023). The model estimated the relative growth rate of PLA (*RGR_PLA1_*, d^-1^), while the initial PLA value (*PLA_Initial_,*cm^-2^) at 0 DAT was assumed to be the same for both treatments, as we homogenised the plant size before the start of the experiment. The fitting procedure had an initial estimate for κ*_b_* = 0.1 and a maximum of 100 iterations. Error handling was implemented using the ‘tryCatch’ algorithm to handle potential convergence issues.

PLA at the end of the photoperiod and dark period, or their equivalent times under CL, were selected to calculate the relative growth rate of projected leaf area (‘*RGR_PLA2_’*, dimensionless) as described in Equation 2.

(2) $$RGR_{PLA2}=\frac{{\text{ln PLA}}_{DAT_{2}}-{\text{ln PLA}}_{DAT_{1}}}{{\text{DAT}}_{2}-{\text{DAT}}_{1}}$$

### Floor coverage and light interception measurements

From 1 to 19 DAT, the percentage of leaf-covered surface area, termed floor coverage (%), was also determined. The light interception (*DLI_INT_*, mol·m^-2^·d^-1^) was calculated per experimental replicate by multiplying the supplied DLI by the floor coverage at each DAT. Light interception showed a sigmoid increase with slight asymmetry; therefore, we chose a Richards function to fit the data (Equation 3) (Heuvelink, 1996; Richards, 1959; Takahashi and Takahashi, 2020).

(3) $$DLI_{Int}=\frac{DLI\_Int_{max}}{{\left(1+e^{-RGR_{DLI\_int}\left(DAT-t_{m}\right)}\right)}^{\frac{1}{v}}}$$

Where *DLI_Int_max_*, was the maximum light interception (mol·m^-2^ at 19 DAT); RGR_DLI_int_ was the relative growth rate of DLI interception (d^-1^); *t_m_* was the inflexion point (d^-1^) at which ‘*RGR_DLI_int_’* was at maximum rate, and *v* corresponded to asymmetry correction (dimensionless). We fitted the model with the ‘brms’ package (Bürkner, 2017), for Bayesian inference via Hamiltonian Monte Carlo (HMC) sampling, with ‘Stan’ package (Carpenter et al., 2017) in R software. All priors, ~N(µ, σ), were set to a normal distribution (N), and µ and σ represented the mean and standard deviation, respectively. Priors were parametrised as follows: ‘*DLI_Int_max_*’ ~ N (16.9, 0.25), ‘*RGR_DLI_int_*’ ~ N (0.5, 0.3), ‘*t_m_*’ ~ N (13, 6), and ‘*v*’ ~N(2, 1). The model used a Gaussian likelihood function, and four Markov Chain Monte Carlo (MCMC) chains were run with 4000 iterations each, including a warm-up phase of 1000 iterations per chain. To ensure efficient sampling, the control parameter ‘adapt_delta’ was set to 0.95 to prevent divergent transitions. Lastly, for ‘Danstar’ and ‘Jagger’, the cumulative light interception was calculated by integrating daily light interception from 1 to 19 DAT for each experimental replicate.

### Measured variables at harvest

Destructive measurements were conducted on 19 DAT. Shoot fresh mass (g · plant^-1^) was measured after discarding the roots (sliced at stone wool block surface). Leaves were manually counted (leaf blade length ≥ 5 mm) and scanned to quantify leaf area (cm²·plant^-1^) using a leaf area meter (LI-3100, LI-COR, Lincoln, NE - USA). Shoot dry mass (g · plant^-1^) was determined by drying harvested shoots in a ventilated oven (Elbanton Special Products by Hettich Benelux, Geldermalsen, The Netherlands) for 24 h at 70°C followed by 48 h at 105°C, following the methodology of Jin et al. (2023b); Susilo et al. (2025); Yan et al. (2019a). The dry matter content was calculated as a percentage, representing the ratio of dry to fresh mass. The leaf area index (LAI) consisted of the leaf area per unit of ground area (surface), and the specific leaf area was the leaf area per unit of shoot dry mass. Light use efficiencies (LUE) were calculated as the mass (fresh or dry mass) per square meter divided by the cumulative light interception.

### Quantification of non-structural carbohydrates by HPLC

Following leaf area measurements, two leaf discs (21 mm diameter) were sampled (at noon, corresponding to the midpoint of the 18-hour photoperiod) from the largest leaf of each plant, snap-frozen in liquid nitrogen, and stored at -80°C. Next, leaf tissue was lyophilised at -60°C for 72 hours (CHRIST - Alpha 1–4 LD plus - Germany). Tissue was ground to a powder using a mixer mill (Retsch MM400, Retsch, Haan - Germany). Ground tissue (150 mg) was diluted in 5 mL of ethanol (80% v/v) to extract glucose, fructose, and sucrose from the soluble fraction (**Supplementary Section A**). After extraction, the remaining tissue (insoluble fraction containing starch) was rinsed three times with 80% ethanol, then enzymatically digested to convert starch into glucose (**Supplementary Section B**). Carbohydrate contents were quantified using high-performance liquid chromatography (HPLC) (DionexTM ICS-5000, Thermo Scientific) equipped with an analytical column (Dionex CarboPacTM PA1, BioLCTM, 2 × 250 mm, Thermo Scientific).

### Energy use and energy use efficiency estimation

Energy use for climate control strategies was estimated using a dynamic, mechanistic model (Sooderjani et al., 2025). Model inputs included PPFD, photoperiod, average air temperature and relative humidity, air speed, and CO_2_ supply, consistent with the growth experiment. In addition, the model incorporated growth chamber dimensions (cultivation area), number of layers, and air-duct design. The climate chamber was assumed to be airtight and well-insulated, with perfectly mixed air in each growth compartment.

The model yielded estimates of energy use for the lighting system (LED energy use), the air conditioning system (HVAC energy use), and their sum, referred to as total energy use (Total energy use). Three scenarios were defined. In scenario A, both the 18-hour photoperiod and CL treatments were set at the same photon efficacy of the LEDs (3.6 µmol·J^-1^), assuming that photon efficacy is independent of PFD or, alternatively, that the lower PFD was achieved by reducing the number of LED fixtures in the system while maintaining identical fixture efficacy. Scenario B assumes that the lower photon efficacy in the CL treatments is achieved by decreasing the drive current in each LED fixture and not by decreasing the number of LED fixtures within the system. Based on the assumption that the lower drive current enables higher photon efficacy, scenario B assigned a photon efficacy of 3.4 µmol·J^-1^ to the 18-hour photoperiod and 3.6 µmol ·J^-1^ to CL (Kusuma et al., 2020; Stanghellini and Katzin, 2024). Scenario C combined the custom photon efficacies from scenario B with a variable temperature regime: 24°C during the 18-hour photoperiod and 20°C during the 6-hour dark period, resulting in the same average daily temperature as the other scenarios (23°C). The same temperature regime was also applied to the CL treatment. This day–night temperature differentiation was designed to resemble temperature management practices (Chen et al., 2022; Pennisi et al., 2020a, 2020b; Tarr and Lopez, 2025). The resulting average air temperature (23°C) matched the average temperature in the growth experiment.

The coefficient of performance (COP) of the air conditioning system, a dimensionless measure of efficiency, was set at 3 (Kozai et al., 2020; Miserocchi and Franco, 2025). Simulated energy use was expressed in kilowatt-hours per square meter of growing area (kWh·m^-2^).

Energy use efficiency (EUE) was calculated as the ratio of measured fresh or dry mass (g·m^-2^) to energy use, expressed separately for the LED system (EUE LED), the HVAC system (EUE HVAC), and their combined total (EUE LED + EUE HVAC). EUE values were reported in g·kWh^-1^ for both fresh and dry mass.

### Experimental design and statistical analysis

The experiment was a split-plot design with four independent experimental replicates in time (blocks), with photoperiod (18-hour) or CL as the main factor and cultivar as the split factor. Four of 168 plants were identified as outliers based on Tukey (1977) criterion (Q1 - 1.5·IQR to Q3 + 1.5·IQR), and excluded from further analysis. Outliers were identified within treatment × cultivar × replicate blocks (42 plants per block; 18 h/CL × ‘Danstar’/’Jagger’), leading to the exclusion of one plant from replicate #2 (Danstar, CL), one from replicate #3 (Jagger, 18 h), and two from replicate #4 (Danstar, 18 h).

A split-plot analysis of variance with blocks was conducted for each variable, including the parameter estimates from the exponential and Richards models. The normality of residuals was assessed using a Shapiro–Wilk test, and equal variance was assumed as only four replicate experiments did not allow for sound testing of this assumption. To separate means, Fisher’s protected LSD test was used at *p = 0.05*. These statistical tests were conducted in Genstat software (19th edition, VSN International, London, UK), and data visualisation was prepared in R software (R Core Team, 2023) using the ‘ggplot2’ package (Wickham, 2016).

## Results

### CL did not disrupt the diurnal increase in projected leaf area

**Figure 1:**
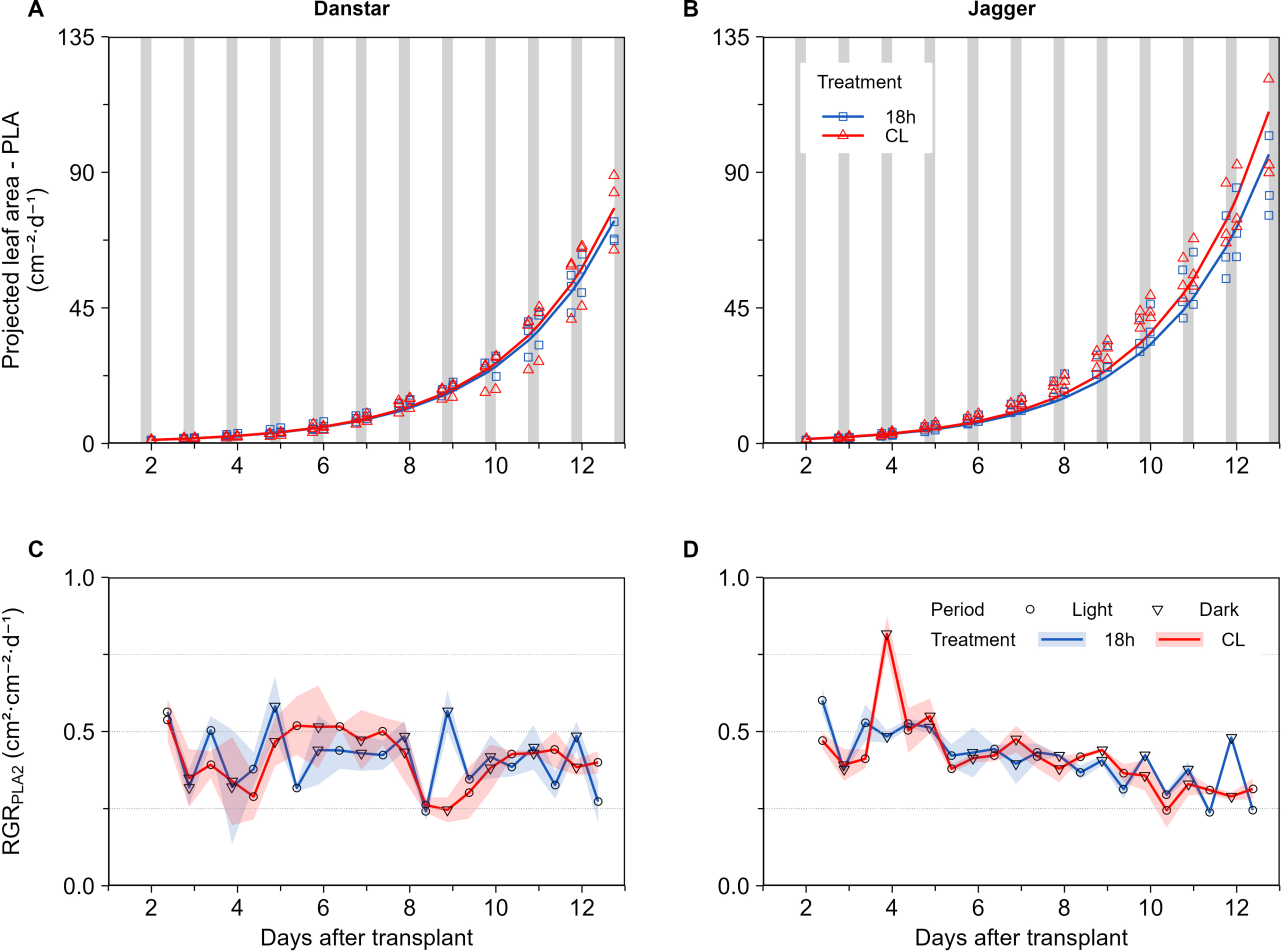
Effect of continuous light (CL) and the 18-hour photoperiod (same DLI: 16.9 mol·m^-2^·d^-1^) on projected leaf area (PLA) and relative growth rate of projected leaf area (RGR_PLA2_) of ‘Danstar’ **(A, C)** and ‘Jagger’ **(B, D)**. Markers represent the means based on three independent replicates (blocks), each consisting of 5 to 9 plants. The shaded region around the lines **(C, D)** represents the standard error of the means (SEM). White and grey areas denote the photoperiod and dark period, respectively, under the 18-hour photoperiod treatment.

From 2 to 13 DAT, CL did not affect projected leaf area (PLA) compared with the 18-hour photoperiod (**Figures 1A, B**; **Supplementary Table S1**). Regardless of treatment or cultivar, the relative growth rate of projected leaf area (RGR_PLA2_) was similar between photoperiod and dark period (**Figures 1C, D**).

### CL enhanced light interception through leaf morphology, in a cultivar-dependent manner

CL altered LAI in a cultivar-dependent manner, with a 23% increase in ‘Jagger’, and a non-significant 6% increase in ‘Danstar’ compared with the 18-hour photoperiod (**Table 2**). Averaged over both cultivars, CL marginally reduced the specific leaf area (SLA) by 2% compared with the 18-hour photoperiod (**Table 2**). Leaf number in ‘Danstar’ and ‘Jagger’ was similar across the 18-hour photoperiod and CL (**Supplementary Table S2**).

**Table 2:** Effect of continuous light (CL) and the 18-hour photoperiod (same DLI of 16.9 mol·m^-2^·d^-1^), on shoot fresh mass, shoot dry mass, shoot dry matter content, leaf area index (LAI), and specific leaf area (SLA) at 19 days after transplant (DAT) for lettuce cultivars ‘Danstar’ and ‘Jagger’.

Treatment	Shoot fresh mass (g · plant^-1^)	Shoot dry mass (g · plant^-1^)	Dry matter content (%)	LAI (cm²·cm^-2^)	SLA (cm²·g^-1^)
Photoperiod (averaged over two cultivars)					
18-hour	45.0 b	2.02 b	4.78 b	4.2	369 a
CL	51.9 a^iii^	2.38 a	4.94 a	4.9	361 b
SEM ^ii^	0.75	0.04	0.02	0.05	2
*P-value ^i^*	*0.008*	*0.005*	*0.009*	*0.003*	*0.035*
Photoperiod × Cultivar					
18-hour × Danstar	40.6	2.00	5.23	3.1 c	279
CL × Danstar	42.9	2.27	5.62	3.3 c	257
18-hour × Jagger	49.5	2.04	4.33	5.3 b	460
CL × Jagger	60.9	2.50	4.26	6.5 a	465
SEM ^ii^	1.88	0.07	0.09	0.15	5
*P-value ^i^ (Interaction)*	*0.11*	*0.25*	*0.11*	*0.038*	*0.099*

^i^*P-values* represent the F-probability for treatment effect in a split-plot analysis of variance: photoperiod (main-plot) and photoperiod × cultivar interaction.

^ii^ SEMs (standard error of the treatment means) for main-plot and interaction levels. SEMs are based on common variance and calculated from four independent replicates (each with 10 to 11 plants per cultivar).

^iii^ Means followed by different letters indicate significant differences at main-plot and interaction levels according to Fisher’s protected LSD test (*p = 0.05*).

Values represent treatment means based on four independent replicates, each consisting of 10 to 11 plants per cultivar.

**Figure 2:**
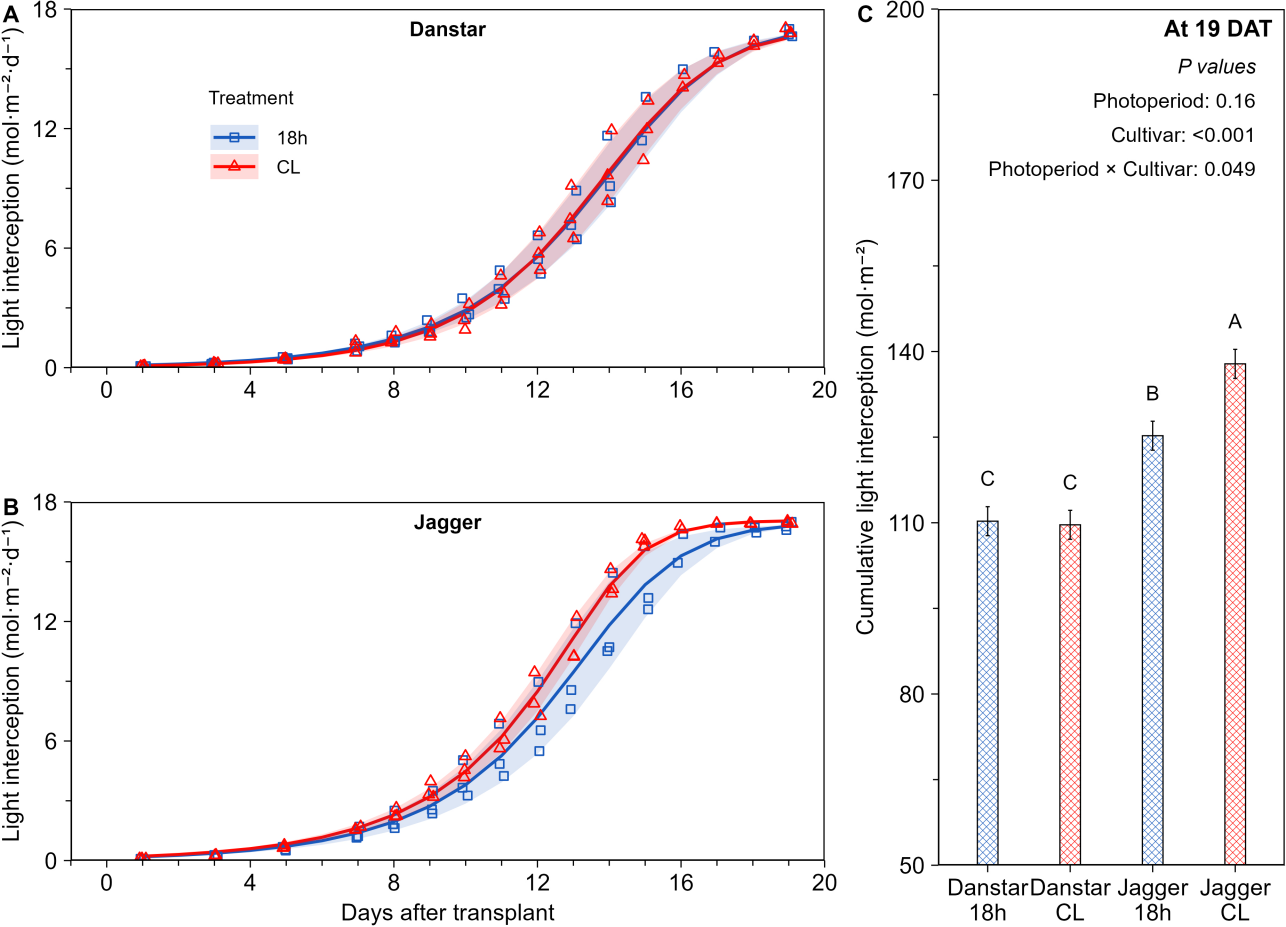
Effect of continuous light (CL) and the 18-hour photoperiod (same DLI of 16.9 mol·m^-2^·d^-1^), on light interception of ‘Danstar’ **(A)** and ‘Jagger’ **(B)**, lines represent the fitted model, shaded areas represent the standard error of the means (SEM). Markers are the average based on three independent replicates (n = 5 to 9 plants per replicate). Shaded regions around the lines represent the standard error of the means (SEM). Effect of photoperiod × cultivar interaction on the cumulative DLI interception at 19 DAT **(C)**, bars with different letters indicate significant differences at the photoperiod × cultivar interaction level according to Fisher’s protected LSD test (*p = 0.05*), error bars represent the SEM.

In ‘Danstar’, CL did not affect light interception (**Figures 2A, C**; **Supplementary Table S3**). Whereas, in ‘Jagger’, CL progressively increased light interception over time (DAT) (**Figure 2B**; **Supplementary Table S3**), compared with the 18-hour photoperiod. At 19 DAT, CL increased the cumulative light interception of ‘Jagger’ by 10% compared with the 18-hour photoperiod (**Figure 2C**).

### CL enhanced lettuce growth, with cultivar-dependent increases in fresh and dry mass

Averaged over both cultivars, continuous light (CL) increased the shoot fresh mass by 15% and shoot dry mass by 18% compared with the 18-hour photoperiod. The slightly higher increase in shoot dry mass than in fresh mass was related to a marginal 3% increase in shoot dry matter content compared with the 18-hour photoperiod (**Table 2**).

Under CL, ‘Danstar’ exhibited a slight 6% higher shoot fresh mass and a 14% higher shoot dry mass compared with the 18-hour photoperiod. ‘Jagger’, in contrast, showed a 23% higher shoot fresh and dry mass compared with the 18-hour photoperiod. CL effects on shoot fresh and dry mass led to a 7% increase in shoot dry matter content in ‘Danstar’, and it was unaffected in ‘Jagger’ compared with the 18-hour photoperiod.

### At equal DLI, continuous light enhances light-use efficiency in lettuce

**Figure 3:**
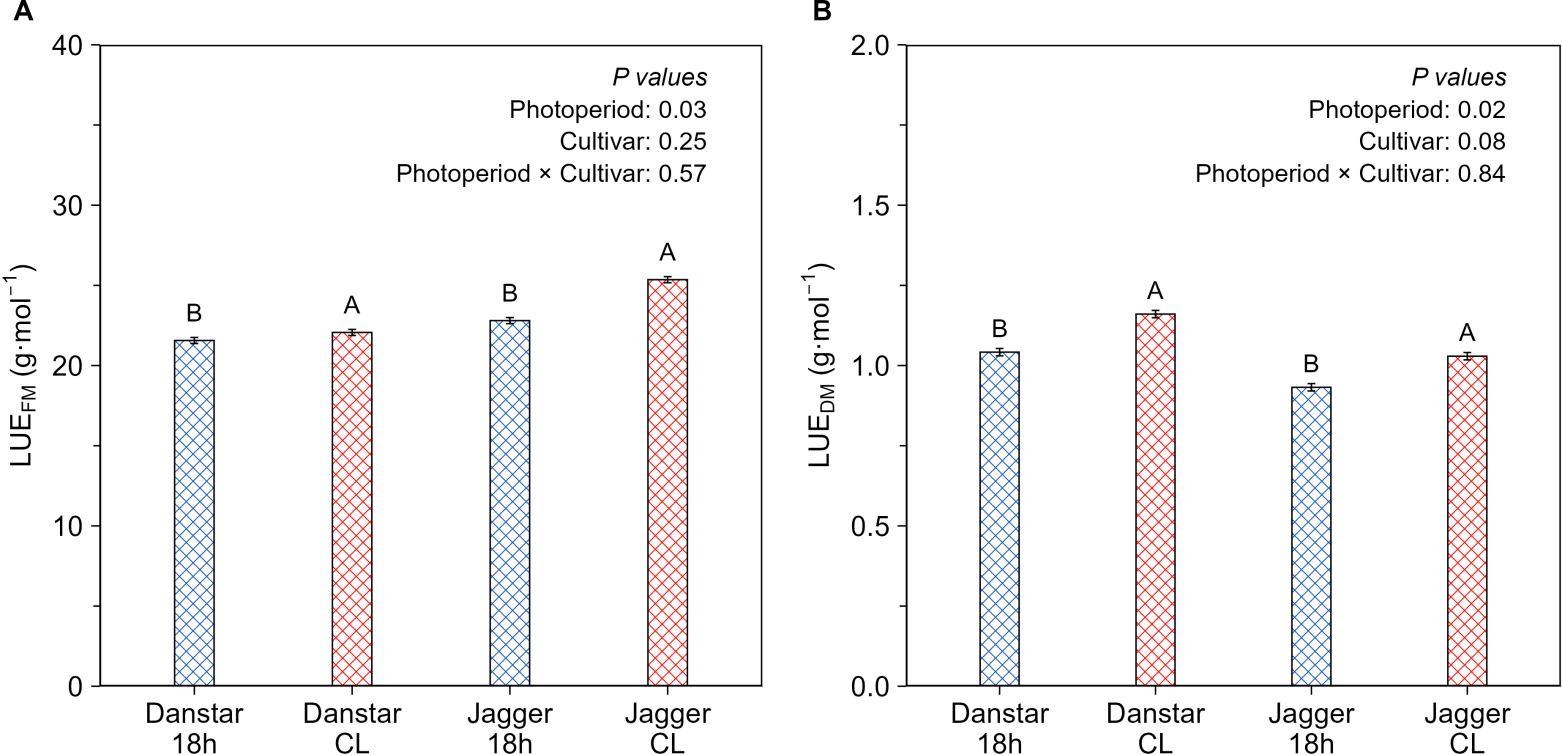
Effect of continuous light (CL) and the 18-hour photoperiod (same DLI of 16.9 mol·m^-2^·d^-1^) on fresh (LUE_FM_) and dry mass (LUE_DM_) based light use efficiency (LUE) of ‘Danstar’ and ‘Jagger’, **(A, B)** LUE was calculated as ratio of shoot fresh or dry mass to cumulative intercepted light. No significant interaction photoperiod × cultivar was observed. Different letters indicate significant differences (Fisher’s protected LSD test (*p = 0.05*) between CL and the 18-hour photoperiod. Error bars represent the standard error of the means (SEM) calculated at photoperiod level.

Averaged over both cultivars, CL increased the fresh mass-based light use efficiency (LUE_FM_; ratio shoot fresh mass to cumulative intercepted light) by 7% and the dry mass-based light use efficiency (LUE_DM_) by 11% compared with the 18-hour photoperiod. Although there was no photoperiod × cultivar interaction on LUE for either fresh or dry mass, nor was there an effect of cultivar (**Figures 3A, B**) the increase in LUE_FM_ was on average larger in Jagger (11%) than in Danstar (2%).

### CL alters sucrose and starch content but not hexose content

At 19 DAT, CL reduced the sucrose content (averaged over cultivars) by 15% compared with the 18-hour photoperiod. In contrast, CL increased the starch content by 12% in ‘Danstar’ and by 49% in ‘Jagger’ relative to the 18-hour photoperiod. Averaged over both cultivars, CL did not affect the glucose content compared with the 18-hour photoperiod and reduced fructose content by 14%, though the effect was not statistically significant (**Table 3**).

**Table 3:** Effect of continuous light (CL) and the 18-hour photoperiod (same DLI of 16.9 mol·m^-2^·d^-1^), on glucose, fructose, sucrose and starch (reported as glucose) in ‘Danstar’ and ‘Jagger’ leaves at 19 days after transplant (DAT).

Treatment	Glucose (mg · g_DW_^-1^)	Fructose (mg · g_DW_^-1^)	Sucrose (mg · g_DW_^-1^)	Starch (mg · g_DW_^-1^)
Photoperiod (averaged over two cultivars)				
18-hour	21.8	33.2	40.9 a^iii^	61.8 b
CL	21.8	28.6	34.6 b	80.0 a
SEM^ii^	1.5	1.6	1.1	1.6
P-value^i^	0.99	0.13	0.03	0.004
Photoperiod × Cultivar				
18-hour × Danstar	28.7	41.4	42.2	65.4
CL × Danstar	24.6	31.8	31.4	73.5
18-hour × Jagger	14.8	25.0	39.7	58.2
CL × Jagger	19.0	25.3	37.9	86.6
SEM ^ii^	2.5	3.0	2.2	3.4
P-value ^i^ (Interaction)	0.20	0.22	0.14	0.052

^i^*P-values* represent the F-probability for treatment effect in a split-plot analysis of variance: photoperiod (main-plot) or photoperiod × cultivar interaction.

^ii^SEMs (standard error of the treatment means) for main-plot or interaction levels. SEMs are based on common variance and calculated from four independent replicates (each with 10 to 11 plants per cultivar).

^iii^Means followed by different letters indicate significant differences at main-plot level according to Fisher’s protected LSD test (*p = 0.05*).

Values represent treatment means based on four independent replicates, each consisting of 9 to 11 plants per cultivar.

### CL does not change energy use and enhances the growth-related energy use efficiency

Simulations conducted under Scenarios A to C quantified the effects of CL on LED, HVAC, and total energy use relative to the 18-hour photoperiod (**Table 4**). Assuming equal photon efficacy of the LEDs, as in Scenario A, LED energy use was the same between treatments. However, the slightly lower HVAC energy use under CL (−4%) marginally reduced total energy use by 1% compared with the 18-hour photoperiod.

**Table 4:** Simulated LED (lighting), HVAC (air conditioning) and total energy use for the 18-hour photoperiod and CL treatments (same DLI of 16.9 mol·m^-2^·d^-1^, 0 to 19 DAT).

Treatment	Energy use (kWh·m^-2^)^*^								
	Equal photon efficacy of LEDs			Custom photon efficacy of LEDs					
	T_light_ = T_dark_ (Scenario A)^**^			T_light_ = T_dark_ (Scenario B)			T_light_ > T_dark_ (Scenario C)		
	LED	HVAC	Total	LED	HVAC	Total	LED	HVAC	Total
18-hour photoperiod	31.0	9.6	40.6	32.8	10.2	43.0	32.8^***^	10.5	43.4
CL	31.0	9.2	40.2	31.0	9.2	40.2	31.0^***^	9.3	40.3
CL vs. 18-hour phot. % change	0%	-4%	-1%	-6%	-10%	-6%	-6%	-11%	-7%

*Energy use values cumulated up to 19 DAT.

**Temperatures: T_light_ during photoperiod, T_dark_ during dark period, or equivalent periods under CL.

***Similar LED energy use as scenario B.

Equal photon efficacy of LEDs = 3.6 µmol ·J^-1^.

Custom photon efficacy of LEDs: CL = 3.6 µmol ·J^-1^ and 18-hour photoperiod = 3.4 µmol ·J^-1^.

*Scenario A:* equal photon efficacy of the LEDs (3.6 µmol ·J^-1^) and constant air temperature 23 °C. *Scenario B*: Custom photon efficacy of the LEDs (3.4 µmol ·J^-1^ for the 18-hour photoperiod and 3.6 µmol ·J^-1^ for CL) with constant air temperature (23 °C). *Scenario C*: Air temperature: T_light_ > T_dark_ (24 °C/20 °C during photoperiod/dark period in the 18-hour photoperiod (or equivalent periods under CL), and custom photon efficacy of the LEDs as in Scenario B.

When a higher photon efficacy of the LEDs was assumed under CL (3.6 µmol ·J^-1^) compared with the 18-hour photoperiod (3.4 µmol ·J^-1^), as in scenario B, LED energy use decreased by 6%, accompanied by a 10% decrease in HVAC energy use, resulting in overall 6% decrease in total energy consumption relative to the 18-hour photoperiod.

In Scenario C, the simulation incorporated a variable temperature regime of 24°C during the photoperiod (T_light_) and 20°C during the dark period (T_dark_) or equivalent periods under CL, alongside the custom photon efficacy of the LEDs simulated in Scenario B. Under these conditions, LED energy use was reduced by 6% under CL. Combined with an 11% lower HVAC energy demand, this resulted in a 7% decrease in total energy use compared with the 18-hour photoperiod.

Total energy use efficiency (EUE) was consistently higher under CL compared with the 18-hour photoperiod across all scenarios. In scenario A (**Table 5**), EUE increases were 16% on a fresh-mass basis (+15% LED, + 20% HVAC) and 18% on a dry mass basis (+19% LED, + 23% HVAC). While in scenario B (**Supplementary Table S4**), EUE increased by 23% on a fresh mass basis (+22% LED, + 27% HVAC), and by 26% on a dry mass basis (+31% LED, + 27% HVAC). In scenario C (**Supplementary Table S5**), the EUE improvements were largest, with increases of 24% on a fresh mass basis (+22% LED, + 30% HVAC) and 27% on a dry mass basis (+26% LED, + 34% HVAC).

**Table 5:** Energy use efficiency (EUE) under continuous light (CL) and the 18-hour photoperiod (same TPFD of 20.1 mol·m^-2^·d^-1^; 0 to 19 DAT).

Treatment	Energy use efficiency (Fresh mass based) (g_FM_ · kWh^-1^)			Energy use efficiency (Dry mass based) (g_DM_ · kWh^-1^)		
	LED	HVAC	Total	LED	HVAC	Total
Photoperiod (averaged over two cultivars)						
18-hour	81.4	263.4	62.2	3.6	11.8	2.8
CL	93.6	316.4	72.2	4.3	14.5	3.3
Photoperiod × Cultivar						
18-hour × Danstar	73.3	237.4	56.0	3.6	11.7	2.8
CL × Danstar	77.4	261.5	59.7	4.1	13.8	3.2
18-hour × Jagger	89.4	289.4	68.3	3.7	11.9	2.8
CL × Jagger	109.9	371.4	84.8	4.5	15.3	3.5
CL vs. 18-hour phot. % change	15%	20%	16%	19%	23%	18%

EUE was calculated as the ratio of measured shoot fresh or dry mass to simulated energy use under equal photon efficacy of the LEDs: 3.6 µmol ·J^-1^ for both treatments (Scenario A).

## Discussion

### CL promotes leaf area growth and light interception

We hypothesised that, at equal DLI, CL would disturb leaf area growth, reducing projected leaf area (PLA), light interception, and growth. Contrary to expectations, CL slightly improved leaf area growth in ‘Danstar’ and strongly in ‘Jagger’.

No differences were detected between photoperiod and dark period in the relative growth rate of PLA (**Figures 1C, D**). This finding contrasts with the general pattern reported across plant species, where leaf area growth is typically enhanced during the night due to circadian regulation, carbon allocation, and turgor-driven growth (Apelt et al., 2017; King, 1988; Pantin et al., 2011; Takami et al., 1982). In ‘Jagger’, CL promoted greater floor coverage and therefore enhanced light interception compared with the 18-h photoperiod, ultimately resulting in ~10% higher cumulative interception at harvest (**Figures 2B, C**). By contrast, ‘Danstar’ did not exhibit a comparable response, underscoring cultivar differences in adaptation to CL (**Figures 2A, C**). Since leaf number was unaffected (**Supplementary Table S2**), the increased interception (leaf area per leaf) is explained by larger individual leaves, which increased modestly in ‘Danstar’ (~3%) but much more strongly in ‘Jagger’ (~18%) under CL (**Supplementary Table S2**). This increase in leaf area, despite a minor 2% reduction in SLA, aligns with the observed improvement in light interception. These results agree with previous studies showing greater light interception with extended photoperiods (up to 20-hours) or increased leaf width, length and diameter under CL (Jeong et al., 2025; Kelly et al., 2020; Palmer and van Iersel, 2020). Our findings add temporal detail by linking daily PLA (0–19 DAT) directly to light interception and reveal cultivar-specific differences in the functional effects of CL at equal DLI.

Negative effects of CL, such as reduced leaf area growth, altered morphology, necrosis, or chlorosis, have been reported in other species such as tomato, eggplant, cucumber, and onion (Liu and Liu, 2022; Velez-Ramirez et al., 2011), underlining the species- and cultivar-specific nature of responses. Together, these results show that CL did not reduce leaf area growth at equal DLI but even enhanced leaf expansion and light interception in responsive cultivars, underscoring genetic plasticity in lettuce responses to CL.

### CL enhances lettuce growth

To maintain the same DLI, PPFD must be adjusted as the photoperiod changes. In our study at equal DLI (16.8 mol·m^-2^·d^-1^), CL increased the fresh and dry mass of ‘Jagger’ by 23%, while in ‘Danstar’, fresh mass rose by 6% and dry mass by 14% compared with the 18-hour photoperiod (**Table 2**). The growth response of ‘Jagger’ under CL aligns with reports in other cultivars exposed to long versus short photoperiods at the same DLI (Kelly et al., 2020; Koontz and Prince, 1986). In contrast, the smaller gain in ‘Danstar’ fresh mass reflects increased dry matter content in this cultivar (**Table 2**).

Across studies, the growth benefit of CL scales with DLI: minimal at low DLI (4 to 9% increase at 10 mol·m^-2^·d^-1^), moderate at intermediate DLI (20 to 25% at 15 mol·m^-2^·d^-1^), and substantial at high DLI, fresh mass 18 to 47%, dry mass 28 to 55% at 22 mol·m^-2^·d^-1^) compared to a 16-hour photoperiod (Kelly et al., 2020; Koontz and Prince, 1986). At higher DLI, CL tends to increase dry mass more than fresh mass compared with a shorter photoperiod, as we also observed in one out of two cultivars. Even under cycle lengths of 20- to 28-hours length, extended photoperiods slightly enhanced growth of our cultivars (9 to 14%) at the same DLI (14.4 mol·m^-2^·d^-1^) (Núñez Ocaña et al., 2025). Unlike three of the five cultivars tested in Koontz and Prince (1986) that developed tip-burn under CL, our cultivars showed no tip-burn, quality defects or bolting, likely due to breeding for tip-burn resistance and delayed flowering.

Overall, at equal DLI, the magnitude of CL-induced growth depends on the cultivar and is further influenced by DLI and cultivar-specific dry matter content. Our findings indicate that CL at equal DLI can improve CEA profitability by achieving equal or higher yields (**Table 2**) with lower light intensity (reduced investment in lighting).

### CL induces starch but not sucrose accumulation

We hypothesised that, at equal DLI, CL compared with the 18-hour photoperiod would alter carbohydrate metabolism, leading to increased sucrose and starch contents in lettuce leaves. Contrary to expectation, while CL did increase the starch by 30% it reduced sucrose by 15%, while glucose and fructose remained unchanged relative to the 18-hour photoperiod (**Table 3**). At 14.3 mol·m^-2^·d^-1^ DLI, CL also lowered sucrose in ‘Jagger’ by 15% relative to the 18-hour photoperiod, but starch rose only 13% (Núñez Ocaña et al., 2025).

These results contrast with earlier studies reporting increased sucrose and starch under CL compared to other photoperiods (Haque et al., 2015; Ohtake et al., 2018; Velez-Ramirez et al., 2011). ‘Jagger’ at 16.8 mol·m^-2^·d^-1^ produced 8% less sucrose and 25% more starch under CL compared to CL at 14.3 mol·m^-2^·d^-1^ (Núñez Ocaña et al., 2025), indicating that carbohydrate responses do not scale proportionally with the 18% increase in DLI. A similar trend occurred under the 18-hour photoperiod, where sucrose decreased by 8% and starch increased by 14% at 16.8 mol·m^-2^·d^-1^ compared to 14.3 mol·m^-2^·d^-1^.

The carbohydrate response to CL appears highly cultivar-dependent. At the same DLI (23 mol·m^-2^·d^-1^), one cultivar showed large increases in sucrose (87%) and starch (190%) compared with the 18-hour photoperiod (Liu and Liu, 2022), whereas another cultivar at the same DLI (25.9 mol·m^-2^·d^-1^) showed decreases of 13% and 4%, respectively, under CL relative to a 12-hour photoperiod (Proietti et al., 2021).

In other species, such as tomato, onion, and pepper, CL has been linked to carbohydrate accumulation, chlorosis, and senescence, often associated with circadian disruption (Demers et al., 1998; Dorais et al., 1996; Liu and Liu, 2022; Van Gestel et al., 2005; Velez-Ramirez et al., 2017, 2011). The higher starch content under CL reflects the absence of the dark period, when starch is normally degraded (Graf et al., 2010; Scialdone et al., 2013). For example, a modest DLI increase from 14.3 to 16.8 mol·m^-2^·d^-1^ (18%) raised starch in ‘Jagger’ by ~25% (Núñez Ocaña et al., 2025), whereas a larger increase from 16.8 to 21.2 mol·m^-2^·d^-1^ (26%) resulted in ~275% more starch in another cultivar (Urairi et al., 2017).

CL has been reported to downregulate photosynthesis (Demers et al., 1998; Globig et al., 1997; Matsuda et al., 2014; Velez-Ramirez, 2014) or to increase leaf carbohydrate levels (Bradley and Janes, 1985; Demers and Gosselin, 2002). Yet, in our study carbohydrate concentrations remained comparable to those in other experiments at lower DLIs. Moreover, long term CL exposure (19 days; 14.4 mol·m^-2^·d^-1^) did not cause carbohydrate accumulation: glucose, fructose, sucrose and starch contents remained stable in ‘Jagger’ across 60-hour sampling (Núñez Ocaña et al., 2025) and in another cultivar across 24-hour sampling (Liu and Liu, 2022). Notably, none of these studies link photosynthesis downregulation to carbohydrate levels.

Together, these results show that CL shifts carbohydrate partitioning toward starch at the expense of sucrose, with the magnitude of the response varying with cultivar and DLI.

### Possible physiological mechanisms underlying growth responses to CL

Although circadian regulation and photosynthesis were not directly measured in this study, the following discussion integrates our phenotypic and biochemical results with established literature to propose plausible mechanisms underlying the observed growth responses.

Sustained carbon assimilation under CL has been proposed from the disrupted rhythmic expression of *CCA/LHY*, based on molecular studies in other species, where *CCA/LHY* fails to repress *CABII*, a gene encoding light-harvesting complex (LHC) proteins (Dodd et al., 2014; Putterill, 2001; Velez-Ramirez, 2014). Under photoperiods and dark periods (e.g., 18-hour photoperiod and 6-hour dark period), sucrose and starch contents typically fluctuate throughout the day; whereas under CL, their levels remain relatively steady (Liu and Liu, 2022; Núñez Ocaña et al., 2025). These fluctuations are driven by the circadian clock, which optimises the timing for carbon assimilation (Graf and Smith, 2011; McClung, 2019). Stable carbon assimilation has also been reported under CL, with only a marginal decline near the end of 12- to 22-hour photoperiods (Silva et al., 2022). Likewise, other photosynthetic traits, *PSII* efficiency, *NPQ*, *NO*, and electron transport rate (ETR), remained stable under extended photoperiods (22-h) (Elkins and van Iersel, 2020b); such long photoperiod might already represent the acclimation responses that could occur under CL.

Acclimation to CL occurs within 8 to 96 hours after transfer from a certain cycle length, as circadian rhythms progressively dampen (Higashi et al., 2016; Wen et al., 2021). Thus, by 19 DAT, lettuce can be considered fully acclimated to CL. In both CL and the 18-hour photoperiod treatments, PFD (photon flux density) remained below the light saturation point, as indicated by previous light response curves in lettuce (Craker and Seibert, 1983; Knight and Mitchell, 1983; Silva et al., 2022). Light response curves reported by Silva et al. (2022) and Jin et al. (2025) suggest a 12.5% higher quantum yield under CL than under the 18-hour photoperiod (0.036 *vs* 0.0032 mol _CO_2__·mol _PFD_^-1^), reflecting more efficient conversion of photons into assimilates under CL, leading to a 13% higher daily leaf carbon assimilation than in the 18-hour photoperiod. Our analysis aligns with previous findings showing that CL or longer photoperiods enhance carbon assimilation compared with shorter photoperiods at the same DLI in lettuce (Kelly et al., 2020; Zhou et al., 2020) and tomato (Haque et al., 2015). This is further supported in our study by consistently higher LUE based on fresh and dry mass under CL relative to the 18-hour photoperiod (**Figure 3A, B**).

In our case, the 13% greater estimated daily leaf carbon assimilation rate under CL, combined with a 10% increase in intercepted light, could help explain the 23% rise in fresh and dry mass observed in ‘Jagger’. By contrast, ‘Danstar’ showed no change in DLI interception but improved daily assimilation under CL, which likely accounts for its substantial 14% increase in dry mass. These findings support our hypothesis that, at the same DLI, CL enhances daily net assimilation via enhanced LUE and quantum yield despite the lower PFD compared with the 18-hour photoperiod. Together, these comparisons suggest that the observed growth responses under CL are consistent with previously reported effects on circadian regulation and photosynthetic efficiency. However, direct measurements of gas exchange and molecular circadian markers are required to confirm these mechanisms.

### CL improves energy use efficiency

The high energy demand for lighting remains a key challenge to sustainable lettuce production in vertical farming (Kong et al., 2019). Efficient lighting in VF depends on both the photon efficacy of the LEDs and the spectral composition of the LED modules (Kusuma et al., 2020). The energy-use model relies on several simplifying assumptions, including airtight and well-insulated growth chambers, perfectly mixed air, a constant coefficient of performance (COP), and ideal LED dimming with stable photon efficacy. In practice, these conditions may vary due to air leakage, spatial heterogeneity, COP dependence on operating conditions, and non-linear LED efficiency responses to drive current. Accordingly, model outputs should be interpreted as relative comparisons among scenarios rather than absolute estimates of energy use.

When both treatments employed enough LED modules to deliver precisely the PFD required to achieve a TPFD of 20.1 mol·m^-2^·d^-1^, and while air temperature and relative humidity (RH) were kept constant, the total energy use was nearly identical (**Table 4**). Under this configuration, the installed LED modules operated at similar photon efficacy, resulting in comparable LED energy use; Maintaining air temperature and RH constant slightly reduced HVAC energy demand in the CL treatment.

If both treatments are assumed to use the same number of LED modules, the modules in the CL treatment require dimming to maintain the target TPFD. This allowed the LEDs to operate at higher photon efficacy, substantially reducing LED energy use (**Table 4**). Combined with the enhanced growth under CL, this configuration markedly enhanced energy use efficiency compared to the 18-hour photoperiod (**Supplementary Table S4**). Importantly, the lower PFD in the CL treatment could also be achieved by reducing the number of LED modules in the system, and this would result in no change in photon efficacy (as in scenario A). However, in this system, the initial investment cost would be lower because fewer LED modules would need to be purchased.

Introducing a temperature differential between the light and the dark period, mimicking commercial production conditions with higher temperatures during photoperiod and lower temperatures during the dark period, slightly decreased HVAC and total energy use further (**Table 4**). This indicates that once the photon efficacy of the LEDs is optimised, adjustments to temperature regimes contribute relatively little to already enhanced energy use efficiency (**Supplementary Table S5**).

Overall, these comparisons show that the primary drivers of energy use are the configuration and operating conditions of the LED modules, while HVAC control strategies play a secondary role. Across all scenarios, the simulated energy use aligns with values reported in other studies (30 to 53 kWh·m^-2^) (Ohyama et al., 2018; Talbot and Monfet, 2024), although those studies typically applied shorter photoperiods (≤ 16 h) and lower PPFD (≤ 200 µmol·m^-2^·s^-1^). Our EUE values (58–85 g_FM_ · kWh^-1^ across treatments and scenarios) are near the lower bound of the typical range reported for vertical farms (80–130 g_FM_·kWh^-1^) (Pennisi et al., 2025). One explanation for our relatively low EUE could be the low planting density used in our experiment (56 plants·m^-2^). A recent vertical farm study has shown that EUE from LEDs increases substantially as planting density rises, for example, from 123 to 680 plants·m^-2^ at comparable crop age (Jadhav et al., 2025). Importantly, our results compare CL and the 18-hour photoperiod at equal TPFD, highlighting that efficiency gains arise from distributing light over time rather than increasing PFD. By contrast, studies where DLI was increased by raising PPFD at constant photoperiod reported enhanced growth but reduced EUE (Keyvan and Roshandel, 2024; Yan et al., 2019b).

Taken together, these results show that at equal DLI, CL improves EUE substantially (**Table 5**), mainly due to enhanced growth, with additional contributions from energy saving due to improved photon efficacy of the LEDs and temperature regime. This underscores the potential of CL under the modelled conditions and assumptions of this study, particularly in tightly controlled vertical farming systems.

## Conclusions

Continuous light (CL) increased lettuce fresh and dry mass compared with the 18-hour photoperiod at the same DLI, with the magnitude of the response being cultivar-dependent, with one showing a 6% and another a 23% increase. CL did not compromise visual quality, as no chlorosis or tip-burn was observed, and it did not negatively affect the leaf growth rate. Instead, CL progressively increased light interception, as confirmed by average leaf area per leaf. The improved growth was driven by two key factors: 1) enhanced floor coverage, enabling up to 10% greater light interception in one cultivar, and 2) increased quantum yield, hence daily carbon assimilation per unit leaf area, which was 13% higher than under the 18-hour photoperiod.

At equal DLI, CL improved energy use efficiency (EUE) by approximately 16 - 18%, primarily due to enhanced growth, with additional contributions from energy savings linked to higher LED photon efficacy and a more efficient temperature regime. These results highlight the potential of CL under the specific experimental and modelling conditions applied here, to increase growth while improving energy use efficiency.

## Data Availability

The raw data supporting the conclusions of this article will be made available by the authors, without undue reservation.

## Supplementary material

Supplementary Figure 1 Average air temperature °C **(A, B)** and relative humidity % **(C, D)** under 18-hour photoperiod and CL treatments. Measurements correspond to 0 to 19 DAT. Coloured markers and lines represent the 18-hour (blue) and CL (red) treatments. Markers represent values integrated for the photoperiod and dark period. Error bars represent the CI 95% calculated from four independent experimental replicates. White and grey regions indicate photoperiod and dark periods under the 18-hour photoperiod or its equivalent under CL.

Supplementary Figure 2 Spectral distribution for the 18-hour photoperiod **(A)** and CL **(B)** treatments. Both spectra were supplemented with far-red radiation, resulting in the same phytochrome photostationary state (PSS = 0.82) and red to far-red ratio (R:FR = 3.5). Spectra were recorded at the beginning and end of each experimental replicate. Coloured lines represent the 18-hour (blue) and CL (red) treatments.

Supplementary Table 1 Effect of continuous light (CL) and the 18-hour photoperiod (same DLI of 16.9 mol·m^-2^·d^-1^) on the *PLA_initial_* and relative growth rate of PLA (*RGR_PLA1_*). Fitted values result from daily measurements of lettuce projected leaf area from 2 to 12 DAT for lettuce cultivars ‘Danstar’ and ‘Jagger’. Values represent treatment means based on four independent replicates, each consisting of 9 to 11 plants per cultivar. ^i^*P-values* represent the F-probability for treatment effect in a split-plot analysis of variance: photoperiod (main-plot), cultivar (split-plot), or photoperiod × cultivar interaction. ^ii^ SEMs (standard error of the treatment means) for main-plot, split-plot or interaction levels. SEMs are based on common variance and calculated from four independent replicates (each with 10 to 11 plants per cultivar). ^iii^ Means followed by different letters indicate significant differences at main-plot and split-plot levels according to Fisher’s protected LSD test (*p* = 0.05). *NA indicates no test due to both treatments received the same seedlings prior to the start of the experiment.

Supplementary Table 2 Effect of continuous light (CL) and the 18-hour photoperiod (same DLI of 16.9 mol·m^-2^·d^-1^), on leaf area, and leaf number at 19 DAT for lettuce cultivars ‘Danstar’ and ‘Jagger’. Values represent treatment means based on four independent replicates, each consisting of 9 to 11 plants per cultivar.^i^*P-values* represent the F-probability for treatment effect in a split-plot analysis of variance: photoperiod (main-plot), cultivar (split-plot) or photoperiod × cultivar interaction. ^ii^ SEMs (standard error of the treatment means) for main-plot, split-plot or interaction level. SEMs are based on common variance and calculated from four independent replicates (each with 10 to 11 plants per cultivar).^iii^ Means followed by different letters indicate significant differences at split-plot and interaction levels according to Fisher’s protected LSD test (*p* = 0.05).

Supplementary Table 3 Effect of continuous light (CL) and the 18-hour photoperiod (same DLI of 16.9 mol·m^-2^·d^-1^) on the parameters of the Richards function describing DLI interception as a function of DAT: relative growth rate of DLI interception (*RGR_DLI_int_*), maximum DLI intercepted (*DLI_Int_max_)*, DAT at maximum ‘*RGR_DLI_int_*’ (*t_m_*), and sigmoid curve shape factor *(v)*. Fitted values were obtained from estimates of DLI interception, calculated using floor coverage measurements from 1 to 19 DAT.

Supplementary Table 4 Energy use efficiency (EUE) under continuous light (CL) and the 18-hour photoperiod (same TPFD of 20.1 mol·m^-2^·d^-1^; 0 to 19 DAT). EUE was calculated as ratio of measured shoot fresh or dry mass to simulated energy use under custom photon efficacy of LEDs: 3.4 µmol·J^-1^ for the 18-hour photoperiod, 3.6 µmol ·J^-1^ for CL (Scenario B).

Supplementary Table 5 Energy use efficiency (EUE) under continuous light (CL) and the 18-hour photoperiod (same TPFD of 20.1 mol·m^-2^·d^-1^; 0 to 19 DAT). EUE was calculated as the ratio of measured shoot fresh or dry mass to simulated energy use under custom photon efficacy of LEDs: 3.4 µmol J^-1^ for the 18-hour photoperiod, 3.6 µmol ·J^-1^ for CL; combined with a temperature regime: 24°C/20°C during the 18-hour photoperiod/6h dark period or equivalent periods under CL (Scenario C).
