# Supplementary material for: Continuous light at fixed daily light integral enhances lettuce growth due to improved light interception and light use efficiency
Source: Front Plant Sci. 2026 Feb 3;17:1756524. doi: 10.3389/fpls.2026.1756524 (PMC12910367; doi:10.3389/fpls.2026.1756524)
Supplement: Supplementary Figure 1 — Average air temperature °C (A, B) and relative humidity % (C, D) under 18-hour photoperiod and CL treatments. Measurements correspond to 0 to 19 DAT. Coloured markers and lines represent the 18-hour (blue) and CL (red) treatments. Markers represent values integrated for the photoperiod and dark period. Error bars represent the CI 95% calculated from four independent experimental replicates. White and grey regions indicate photoperiod and dark periods under the 18-hour photoperiod or its equivalent under CL. [file DataSheet1.pdf]

Continuous light at fixed daily light integral enhances lettuce growth due to improved light interception and light use efficiency

Diego Núñez Ocaña, Homa Esmaeli, Paul Kusuma, Leo F.M. Marcelis and Ep Heuvelink
Wageningen University and Research – Horticulture and Product Physiology
The Netherlands

Supplementary information

Supplementary section A: HPLC method Carbohydrates

To extract soluble sugars, a 15 mg freeze-dried powder sample was mixed with 5 mL 80 % ethanol in a centrifuge tube and placed in an 80 °C shaking water bath for 20 minutes, and then centrifuged at ~8500 RCF, 4 °C for 5 minutes. The supernatant and pellet were used for sugar and starch analysis, respectively. 1 ml supernatant was transferred to an Eppendorf vial for sugar analysis, and the remaining pellet with ethanol in the centrifuge tube was stored in a -20 °C or -80 °C freezer until starch analysis.

The supernatant in the Eppendorf vial was evaporated in the pre-heat vacuum concentrator (SPD2010, Integrated SpeedVac System, Thermo Scientific) at 55 °C, 5.1 Torr for a 30-minute heat time and 2-hour run time. If the organic solvent ethanol was not completely volatilised, another 1-minute heat time and 30-minute run time under the same conditions were applied. The sugars after ethanol volatilisation were suspended in 1 mL Milli-Q water. Then, the samples were placed in an ultrasonic bath for 10 minutes and centrifuged at 10000 rpm, 19 °C for 10 minutes. Sugar suspension was diluted ten times for sugar measurement by high-performance liquid chromatography (HPLC) analysis.

The HPLC (DionexTM ICS-5000, Thermo Scientific) has an analytical column (Dionex CarboPacTM PA1, BioLCTM, 2 × 250 mm, Thermo Scientific) linked to an electrochemical detector. The chromatographic analysis conditions were as follows. The mobile phase was 100 mM NaOH, and the pump was set at a flow rate of 0.250 mL·min^{-1}. The column temperature was 25 °C. The injection was done by an autosampler (DionexTM AS-AP Autosampler, Thermo Scientific), and the injected volume was 10.0 μL. The detection temperature was 25 °C. The measurement duration of each sample was 30 minutes. The samples' peak times of glucose, fructose and sucrose were determined according to the peak times of those in the standard solution. The average retention times for glucose, fructose, and sucrose were 5.6 minutes, 6.3 minutes, and 11.9 minutes, respectively. Quantification of glucose, fructose and sucrose in the samples was determined by standardising the peak areas with the peak areas in the calibration standard solution.

Supplementary section B: HPLC method Starch

For starch determination, the starch was first converted into glucose and the glucose was measured. The remaining supernatant in the centrifuge tubes was removed. To remove the soluble sugars from the pellet, 3 mL 80 % ethanol was added, and the supernatant was removed again after centrifugation at ~8500 RCF, 4 °C for 5 minutes. After repeating this step twice, the pellet was dried in the vacuum concentrator (SPD2010, Integrated SpeedVac System, Thermo Scientific) at 60 °C, 6.1 Torr for a 20-minute heat time and

20-minute run time. If the pellet was not dried, another 20 minutes was applied. The dried pellet was suspended in 2 mL 1 mg·mL^{-1} α-amylase (SERVA) solution, followed by incubating in a shaking water bath at 90 °C for 30 minutes. Then 1 mL 0.5 mg·mL^{-1} amyloglucosidase (Sigma-Aldrich) in 50 mM citrate buffer (pH = 4.6) was added, and the samples were incubated in a shaking water bath at 60 °C for 10 minutes. After centrifugation at ~8500 RCF, 4 °C for 5 minutes, 1 mL supernatant was transferred to an Eppendorf vial. The suspension was centrifuged at 10000 rpm for 15 minutes and diluted twenty times for glucose measurement by HPLC with the above analysis conditions. The measurement duration of each sample was 13 minutes. The average retention time for glucose was 3.0 minutes.

Supplementary tables

Table S1. Effect of continuous light (CL) and the 18-hour photoperiod (same DLI of 16.9 mol·m^{-2}·d^{-1}) on the $PLA_{initial}$ and relative growth rate of PLA (RGR_{PLA1}). Fitted values result from daily measurements of lettuce projected leaf area from 2 to 12 DAT for lettuce cultivars 'Danstar' and 'Jagger'. Values represent treatment means based on four independent replicates, each consisting of 9 to 11 plants per cultivar.

**Treatment**	**$PLA_{Initial}$ (cm^2)**	**RGR_{PLA1} (d^{-1})**
**Photoperiod (averaged over two cultivars)**		
18-hour	0.61	0.388 b[iii]
CL	0.61	0.395 a
SEM[ii]	0	0.001
P-value [i]	*NA **	*0.05*
**Cultivar (averaged over treatments)**		
Danstar	0.54 b	0.389
Jagger	0.67 a	0.394
SEM [ii]	0.03	0.004
P-value [i]	*0.02*	*0.34*
**Photoperiod × Cultivar**		
18-hour × Danstar	0.54	0.387
CL × Danstar	0.54	0.391
18-hour × Jagger	0.67	0.389
CL × Jagger	0.67	0.400
SEM [ii]	0.04	0.004
P-value [i]*(Interaction)*	*1.00*	*0.50*

[i] *P-values* represent the F-probability for treatment effect in a split-plot analysis of variance: photoperiod (main-plot), cultivar (split-plot), or photoperiod × cultivar interaction.
[ii] SEMs (standard error of the treatment means) for main-plot, split-plot or interaction levels. SEMs are based on common variance and calculated from four independent replicates (each with 10 to 11 plants per cultivar).
[iii] Means followed by different letters indicate significant differences at main-plot and split-plot levels according to Fisher's protected LSD test (p = 0.05).
*NA indicates no test due to both treatments received the same seedlings prior to the start of the experiment.

Table S2. Effect of continuous light (CL) and the 18-hour photoperiod (same DLI of 16.9 mol·m^{-2}·d^{-1}), on leaf area, and leaf number at 19 DAT for lettuce cultivars 'Danstar' and 'Jagger'. Values represent treatment means based on four independent replicates, each consisting of 9 to 11 plants per cultivar.

**Treatment**	**Leaf area (cm^2 · $plant^{-1}$)**	**Leaf number**
**Photoperiod (averaged over two cultivars)**		
18-hour	750	22.2
CL	868	22.8
SEM[ii]	9	0.2
P-value [i]	*0.003*	*0.14*
**Cultivar (averaged over treatments)**		
Danstar	571 b	15.5 b
Jagger	1048 a[iii]	29.5 a
SEM[ii]	26	0.2
P-value [i]	*<0.001*	*<0.001*
**Photoperiod × Cultivar**		
18-hour × Danstar	559 c	15.4
CL × Danstar	582 c	15.6
18-hour × Jagger	941 b	29.0
CL × Jagger	1155 a	30.1
SEM [ii]	27	0.3
P-value [i]*(Interaction)*	*0.038*	*0.12*

[i] *P-values* represent the F-probability for treatment effect in a split-plot analysis of variance: photoperiod (main-plot), cultivar (split-plot) or photoperiod × cultivar interaction.

[ii] SEMs (standard error of the treatment means) for main-plot, split-plot or interaction level. SEMs are based on common variance and calculated from four independent replicates (each with 10 to 11 plants per cultivar).

[iii] Means followed by different letters indicate significant differences at split-plot and interaction levels according to Fisher's protected LSD test (p = 0.05).

Table S3. Effect of continuous light (CL) and the 18-hour photoperiod (same DLI of 16.9 mol·m^{-2}·d^{-1}) on the parameters of the Richards function describing DLI interception as a function of DAT: relative growth rate of DLI interception (RGR_{DLI_int}), maximum DLI intercepted (DLI_Int_{max}), DAT at maximum 'RGR_{DLI_int}' (t_m), and sigmoid curve shape factor *(v)*. Fitted values were obtained from estimates of DLI interception, calculated using floor coverage measurements from 1 to 19 DAT.

**Treatment**	**RGR_{DLI_int} (d^{-1})**	**DLI_Int_{max} (mol · m^{-2} · d^{-1})**	**t_m (d)**	**v**
**Photoperiod (averaged over two cultivars)**				
18-hour	0.84	16.9	14.9	2.4
CL	0.92	16.9	14.4	2.6
SEM[ii]	0.05	0.05	0.15	0.1
P-value [i]	*0.32*	*0.87*	*0.15*	*0.4*
**Cultivar (averaged over treatments)**				
Danstar	0.74 b	16.9	15.0 a	2.0
Jagger	1.02 a[iii]	16.9	14.3 b	3.0
SEM [ii]	0.03	0.04	0.17	0.1
P-value [i]	*0.002*	*0.16*	*0.035*	*0.0*
**Photoperiod × Cultivar**				
18-hour × Danstar	0.75 b	16.9	15.2	2.2
CL × Danstar	0.73 b	16.9	14.8	1.9
18-hour × Jagger	0.92 b	16.9	14.5	2.7
CL × Jagger	1.11 a	16.9	14.0	3.3
SEM [ii]	0.05	0.07	0.23	0.1
P-value [i]*(Interaction)*	*0.047*	*0.19*	*0.72*	*0.0*

[i] *P-values* represent the F-probability for treatment effect in a split-plot analysis of variance: photoperiod (main-plot), cultivar (split-plot), or photoperiod × cultivar interaction.
[ii] SEMs (standard error of the treatment means) for main-plot, split-plot or interaction levels. SEMs are based on common variance and calculated from four independent replicates (each with 10 to 11 plants per cultivar).
[iii] Means followed by different letters indicate significant differences at split-plot and interaction levels according to Fisher's protected LSD test (p = 0.05).

Table S4. Energy use efficiency (EUE) under continuous light (CL) and the 18-hour photoperiod (same TPFD of 20.1 mol·m^{-2}·d^{-1}; 0 to 19 DAT). EUE was calculated as ratio of measured shoot fresh or dry mass to simulated energy use under custom photon efficacy of LEDs: 3.4 µmol ·J^{-1} for the 18-hour photoperiod, 3.6 µmol ·J^{-1} for CL (Scenario B).

**Treatment**	**Energy use efficiency (Fresh mass based) (g·kWh⁻¹)**			**Energy use efficiency (Dry mass based) (g·kWh⁻¹)**		
	**LED**	**HVAC**	**Total**	**LED**	**HVAC**	**Total**
**Photoperiod (averaged over 2 cultivars)**						
18-hour	76.8	248.4	58.7	3.4	11.1	2.6
CL	93.6	316.4	72.2	4.3	14.5	3.3
**Photoperiod × Cultivar**						
18-hour × Danstar	69.2	223.8	52.9	3.4	11.0	2.6
CL × Danstar	77.4	261.5	59.7	4.1	13.8	3.2
18-hour × Jagger	84.4	272.9	64.5	3.5	11.2	2.7
CL × Jagger	109.9	371.4	84.8	4.5	15.3	3.5
CL vs. 18-hour phot. % change	22 %	27 %	23 %	26 %	31 %	27 %

Table S5. Energy use efficiency (EUE) under continuous light (CL) and the 18-hour photoperiod (same TPFD of 20.1 mol·m^{-2}·d^{-1}; 0 to 19 DAT). EUE was calculated as the ratio of measured shoot fresh or dry mass to simulated energy use under custom photon efficacy of LEDs: 3.4 µmol ·J^{-1} for the 18-hour photoperiod, 3.6 µmol ·J^{-1} for CL; combined with a temperature regime: 24 °C / 20 °C during the 18-hour photoperiod / 6h dark period or equivalent periods under CL (Scenario C).

**Treatment**	**Energy use efficiency (Fresh mass based) (g_{FM} · kWh⁻¹)**			**Energy use efficiency (Dry mass based) (g_{DM} · kWh⁻¹)**		
	**LED**	**HVAC**	**Total**	**LED**	**HVAC**	**Total**
**Photoperiod (averaged over 2 cultivars)**						
18-hour	76.8	239.6	58.2	3.4	10.7	2.6
CL	93.6	311.6	72.0	4.3	14.3	3.3
**Photoperiod × Cultivar**						
18-hour × Danstar	69.2	215.9	52.4	3.4	10.6	2.6
CL × Danstar	77.4	257.5	59.5	4.1	13.6	3.1
18-hour × Jagger	84.4	263.3	63.9	3.5	10.8	2.6
CL × Jagger	109.9	365.7	84.5	4.5	15.0	3.5
CL vs. 18-hour phot. % change	22 %	30 %	24 %	26 %	34 %	27 %

Supplementary figures

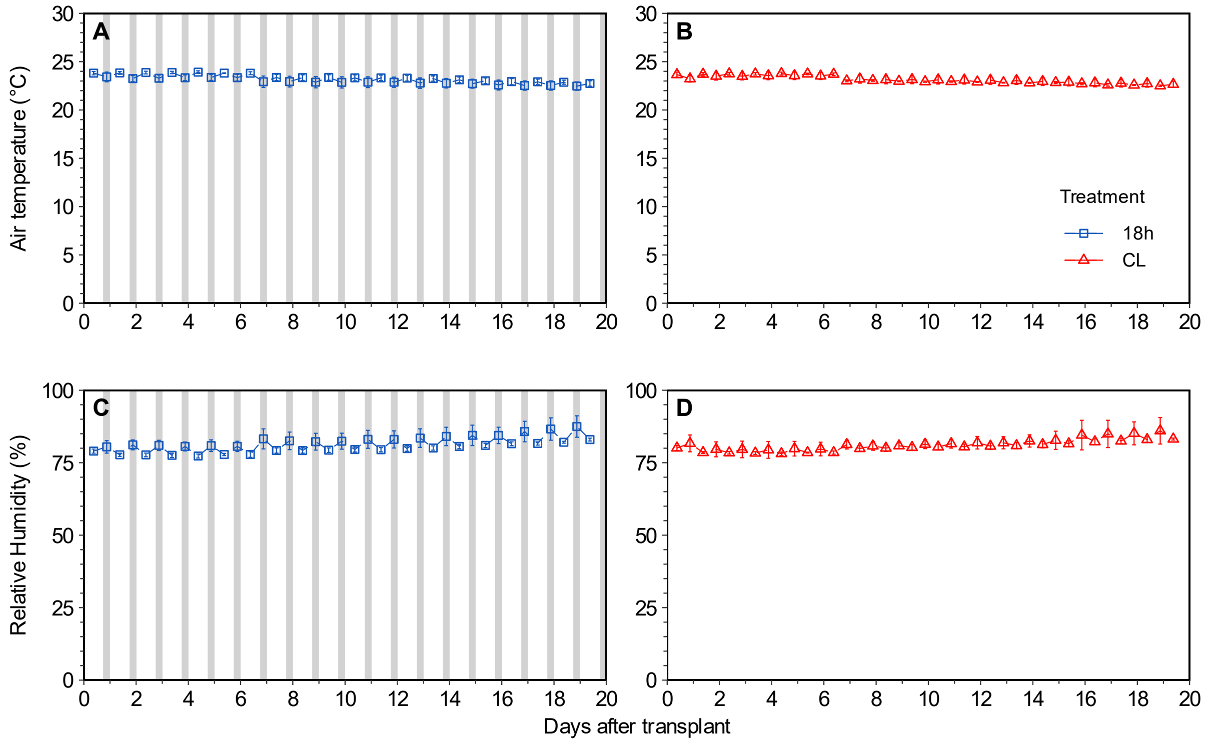

Figure S1. Average air temperature ℃ (A,B) and relative humidity % (C,D) under 18-hour photoperiod and CL treatments. Measurements correspond to 0 to 19 DAT. Coloured markers and lines represent the 18-hour (blue) and CL (red) treatments. Markers represent values integrated for the photoperiod and dark period. Error bars represent the CI 95% calculated from four independent experimental replicates. White and grey regions indicate photoperiod and dark periods under the 18-hour photoperiod or its equivalent under CL.

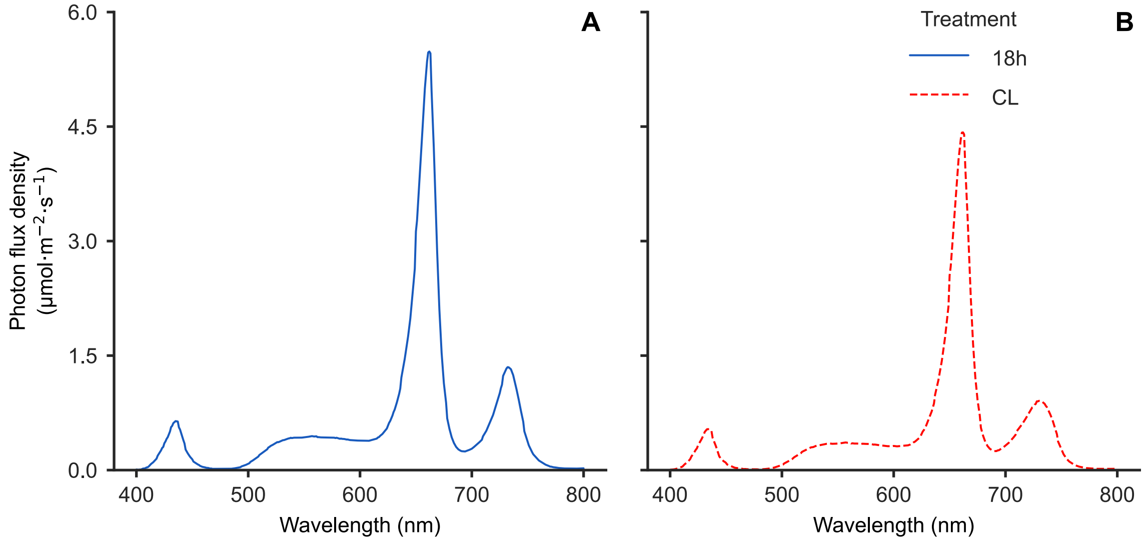

Figure S2. Spectral distribution for the 18-hour photoperiod (A) and CL (B) treatments. Both spectra were supplemented with far-red radiation, resulting in the same phytochrome photostationary state (PSS = 0.82) and red to far-red ratio (R:FR = 3.5). Spectra were recorded at the beginning and end of each experimental replicate. Coloured lines represent the 18-hour (blue) and CL (red) treatments.
